# Streptococcal toxic shock syndrome caused by the dissemination of an invasive emm3/ST15 strain of *Streptococcus pyogenes*

**DOI:** 10.1186/s12879-017-2870-2

**Published:** 2017-12-18

**Authors:** Tsuyoshi Sekizuka, Emina Nai, Tomohiro Yoshida, Shota Endo, Emi Hamajima, Satoka Akiyama, Yoji Ikuta, Natsuko Obana, Takahiro Kawaguchi, Kenta Hayashi, Masahiro Noda, Tomoko Sumita, Masayuki Kokaji, Tatsuo Katori, Masanori Hashino, Kunihiro Oba, Makoto Kuroda

**Affiliations:** 1 Pathogen Genomics Center, National Institute of Infectious Diseases, 1-23-1 Toyama, Shinjuku, Tokyo 162-8640 Japan; 2 Department of Pediatrics, Showa General Hospital, 8-1-1 Hanakoganei, Kodaira, Tokyo 187-8510 Japan

**Keywords:** *Streptococcus pyogenes*, Invasive, *emm3*, ST15, Whole-genome sequencing

## Abstract

**Background:**

*Streptococcus pyogenes* (group A *Streptococcus* [GAS]) is a major human pathogen that causes a wide spectrum of clinical manifestations. Although invasive GAS (iGAS) infections are relatively uncommon, *emm3*/ST15 GAS is a highly virulent, invasive, and pathogenic strain. Global molecular epidemiology analysis has suggested that the frequency of *emm3* GAS has been recently increasing.

**Case presentation:**

A 14-year-old patient was diagnosed with streptococcal toxic shock syndrome and severe pneumonia, impaired renal function, and rhabdomyolysis. GAS was isolated from a culture of endotracheal aspirates and designated as KS030. Comparative genome analysis suggested that KS030 is classified as *emm3* (*emm-*type) and ST15 (multilocus sequencing typing [MLST]), which is similar to iGAS isolates identified in the UK (2013) and Switzerland (2015).

**Conclusions:**

We conclude that the global dissemination of *emm3*/ST15 GAS strain has the potential to cause invasive disease.

**Electronic supplementary material:**

The online version of this article (10.1186/s12879-017-2870-2) contains supplementary material, which is available to authorized users.

## Background

*Streptococcus pyogenes* (group A *Streptococcus*; GAS) is a major human pathogen that causes a wide spectrum of clinical manifestations, from common superficial skin infections and pharyngitis to invasive infections, such as bacteremia, meningitis, cellulitis, and pneumonia, and the more severe necrotizing fasciitis and streptococcal toxic shock syndrome (STSS). Invasive GAS (iGAS) infections, although relatively less common than non-iGAS infections, remain a significant global cause of morbidity and mortality. GAS strains differ widely in the degree of encapsulation, showing a mucoid morphology when cultured on blood agar plates [1]. Limited *emm* types, such as *emm1*, *3*, *5*, *6*, and *18*, which are often mucoid, have been associated with rheumatological effects [2] and are the most prevalent *emm* types found to cause iGAS infections worldwide, particularly *emm1* and *emm3* [3, 4].

## Case presentation

**Fig. 1:**
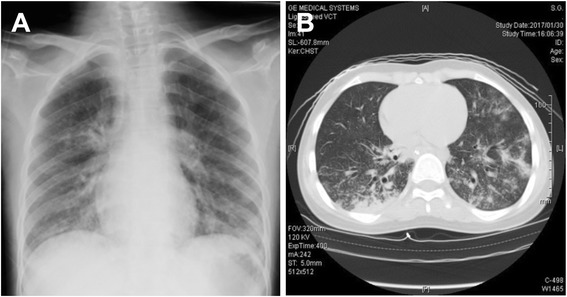
Chest presentation. **a** Chest X-ray on admission, showing pneumonia. **b** Chest CT scan on admission showing consolidation with the air bronchogram

A 14-year-old boy with complaints of fever above 39 °C and sore throat had received an intravenous infusion of fluid and antimicrobial agents for dehydration and bacterial infection at another clinic. Although he had been diagnosed with an immunoglobulin (Ig)G subclass deficiency (IgG4 single deficiency) in the past, he was not considered to be more susceptible to infection. Also, due to mental development delays, he had received special support education. Three days later, he was transported by ambulance to our hospital because of a loss of consciousness. On admission, the Glasgow Coma Scale was E1V1M3, body temperature was 41.7 °C, systolic blood pressure was 74 mmHg, hypotension was observed, SpO_2_ was 80%, and oxygen saturation was markedly reduced. Convulsions began during his treatment in the Emergency Department, so intravenous anticonvulsive drugs were administered. Endotracheal intubation was also performed for the management of ventilation. During the physical examination in the Emergency Department, both ocular conjunctiva were congested and erythema was present across his anterior chest. During chest auscultation, rhonchi were apparent. Chest computed tomography (CT) showed frosted glass shadows on both sides of his back (Fig. 1). There were no abnormal findings on a head CT scan.

Blood test results revealed a white blood cell count of 21,300/μl (neutrophils 83%), platelet count of 20.7 × 10^4^/μl, C-reactive protein level of 33.84 mg/dl, blood urea nitrogen level of 50.9 mg/dl, creatinine level of 1.87 mg/dl, aspartate aminotransferase level of 72 U/L, alanine aminotransferase level of 20 U/L, lactic acid dehydrogenase level of 501 U/L, total bilirubin level of 0.5 mg/dl, gamma-glutamyltransferase level of 14 U/L, creatine kinase level of 2062 U/L, IgG level of 2507 mg/dl, IgM level of 88 mg/dl, and IgA level of 284 mg/dl. Rapid immunochromatographic testing of pharyngeal swabs was positive for GAS antigen. One week before the patient developed a fever, his brother developed a fever with pharyngitis, which was diagnosed as a GAS infection and treated with antimicrobials. Rapid testing results for influenza virus and adenovirus were negative.

Based on the above clinical findings, the patient was diagnosed with STSS and severe pneumonia, impaired renal function, and rhabdomyolysis. Antibiotics (vancomycin, clindamycin, and meropenem) and intravenous immunoglobulin therapy were administered to treat the severe infection. Also, continuous infusion of a vasopressor for hypotension and steroid pulse therapy for the severe pneumonia were administered. Transfusion, anticoagulation therapy, and antithrombin III supplementation therapy were performed because of complications from disseminated intravascular coagulation syndrome during the course of treatment. Due to the prior administration of intravenous antibiotics at the other clinic, his blood culture results were negative, but GAS was isolated from a culture of endotracheal aspirate (the strain was designated KS030). Therefore, the antimicrobial treatment was changed to cefotaxime administration for 14 days. On hospitalization day 3, the patient recovered from STSS, and the pneumonia improved on hospitalization day 9, thus the ventilator was subsequently removed. Rehabilitation was successful, and the patient was discharged on hospitalization day 19 with no sequelae.

**Fig. 2:**
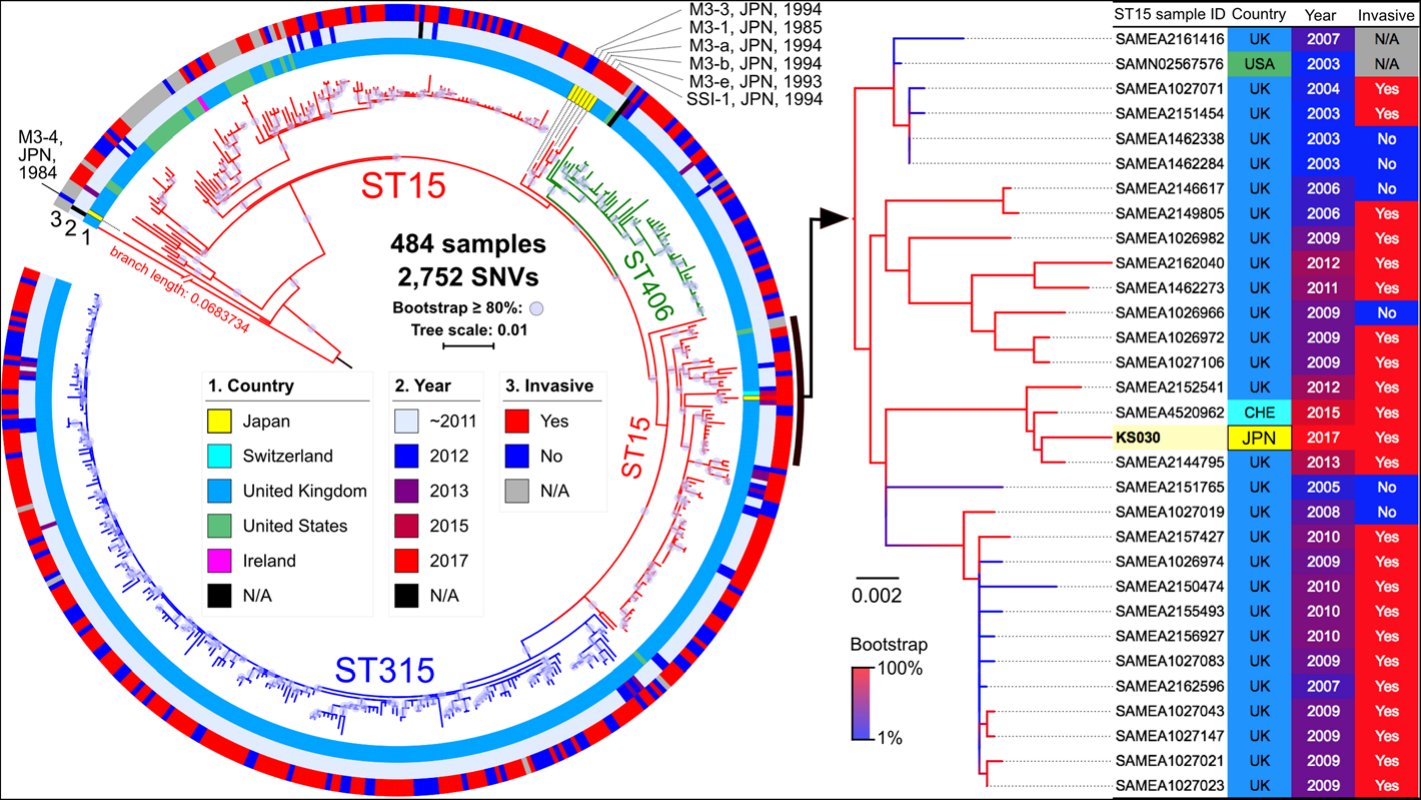
Maximum likelihood phylogeny of the 484 GAS isolates (*emm3*), including KS030, based on 2752 core genome SNVs. *S. pyogenes* KS030 in this study was used as the GAS reference genome sequence

Whole-genome sequencing of *S. pyogenes* KS030 was performed by the hybrid assembly of reads obtained by an Illumina NextSeq 500 sequencer (2 × 150-mer; median coverage: ×218) and a PacBio RSII single-molecule real-time sequencer (N50 read length: 14,559; median coverage: ×279.68). The complete genomic sequence of *S. pyogenes* KS030 was annotated using the gene prediction program Prodigal and deposited in a public database (1,900,008 bp; accession number: AP018337). KS030 is classified as an *emm3* (*emm-*type) and ST15 (multilocus sequencing typing [MLST]) strain that has a similar genomic organization as the *S. pyogenes* SSI-1 strain isolated from an STSS patient in Japan [5]. To characterize the molecular epidemiology of KS030, all publicly available genome sequences of GAS strains, including ST15, ST315, and ST406, were retrieved (see Additional file 1) and compared using bwaMEM read mapping against the KS030 complete genome sequence as a reference. After excluding repeated regions and six prophage sequences throughout the whole genome sequence, 80.3% of the region was assigned as the core genome sequence among 484 GAS strains, which resulted in the identification of a total of 2752 single-nucleotide variations (SNVs). The core genome of MLST (cgMLST) was determined using the above SNVs, and the phylogeny was generated using the maximum likelihood phylogenetic method with RAxML [6]. The results indicated that KS030 is very similar to the iGAS isolate SAMEA2144795 identified in the United Kingdom in 2013 and the SAMEA4520962 isolate identified in Switzerland in 2015 (Fig. 2). The KS030 genome sequence also suggested that the unique 1-bp deletion (7 × to 6 × adenine nucleotides) in the *rocA* gene led to C-terminal truncation, as well as common genetic features among *emm3* GAS isolates (see Additional file 1).

## Discussion and conclusions

The *emm3*/ST15 GAS strain has the potential to be highly virulent, demonstrating invasive pathogenicity [4, 7] and high capsule production, leading to a mucoid morphology [3] and early macrophage cell death [8]. Intriguingly, the molecular epidemiology of GAS in Japan suggests that the frequency of *emm3* GAS has been recently increasing (2%–6.9% in 2010–2012) but is not as dominant as *emm1* (>60%) [9]. Moreover, the natural *rocA* mutation within M3 isolates generates an absence of RocA activity on the CovR/S two-component system resulting in the de-repression of more than a dozen immunomodulatory virulence factors, leading to the severity of invasive infections [10]. We conclude that the global dissemination of *emm3*/ST15 has the potential to cause invasive disease.

## Additional file

Additional file 1: General features of the isolate information and the genome sequences. In addition to KS030 genome sequence, all 483 available draft genome sequences related to ST15 type of *Streptococcus pyogenes* isolates have been retrieved from public short read archive databases, and the all those genome sequences were used for comparative genome analysis as described in the maintext. (XLSX 198 kb)

## Abbreviations

GAS: Group A hemolytic *Streptococcus*
iGAS: Invasive GAS
MLST: Multilocus sequencing typing
SNVs: Single-nucleotide variations
STSS: Streptococcal toxic shock syndrome
